# Infectivity of an Infectious Clone of Banana Streak CA Virus in A-Genome Bananas (*Musa acuminata* ssp.)

**DOI:** 10.3390/v13061071

**Published:** 2021-06-04

**Authors:** Anthony P. James, Dawit B. Kidanemariam, Sharon D. Hamill, James L. Dale, Robert M. Harding

**Affiliations:** 1 Centre for Agriculture and the Bioeconomy (CAB), Queensland University of Technology (QUT), Brisbane, QLD 4001, Australia; ap.james@connect.qut.edu.au (A.P.J.); d.kidanemariam@qut.edu.au (D.B.K.); j.dale@qut.edu.au (J.L.D.); 2 Department of Agriculture and Fisheries (DAF), Maroochy Research Facility, Nambour, QLD 4560, Australia; Sharon.Hamill@daf.qld.gov.au

**Keywords:** badnavirus, banana, banana streak virus, *Caulimoviridae*, infectious clone

## Abstract

We have characterized the complete genome sequence of an Australian isolate of banana streak CA virus (BSCAV). A greater-than-full-length, cloned copy of the virus genome was assembled and agroinoculated into five tissue-cultured plants of nine different *Musa acuminata* banana accessions. BSCAV was highly infectious in all nine accessions. All five inoculated plants from eight accessions developed symptoms by 28 weeks post-inoculation, while all five plants of *M. acuminata* AA subsp. *zebrina* remained symptomless. Symptoms were mild in six accessions but were severe in Khae Phrae (*M*. *acuminata* subsp. *siamea*) and the East African Highland banana accession Igisahira Gisanzwe. This is the first full-length BSCAV genome sequence reported from Australia and the first report of the infectivity of an infectious clone of banana streak virus.

## 1. Introduction

Bananas (*Musa* spp.) are hosts to several badnavirus species referred to collectively as banana streak viruses (BSVs), with nine species currently recognized by the International Committee on Taxonomy of Viruses (ICTV) [1]. BSVs occur in most banana-producing countries where infection typically results in narrow, discontinuous or continuous, chlorotic and necrotic streaks which run parallel to the leaf veins [2]. Symptoms can vary considerably, however, and can also include large chlorotic areas on the leaf lamina, stunting, pseudostem splitting, internal necrosis, cigar leaf necrosis and plant death. Symptom expression is also influenced by a range of factors, including host cultivars, virus species and climatic conditions [3].

BSVs are members of the genus *Badnavirus*, family *Caulimoviridae* which have a double-stranded, circular DNA genome of about 7–9 kbp typically containing three open reading frames (ORFs) [4]. ORF 3 encodes a large polyprotein of ~200 kDa that is processed into several mature functional proteins. The reverse transcriptase (RT)/ribonuclease H (RNase H)-coding sequence of ORF 3 is the most highly conserved region, and a nucleotide sequence difference of more than 20% in this region is used for demarcation of species in the genus [1].

Infectious clones have been reported for several badnaviruses, including the Commelina yellow mottle virus (ComYMV), Citrus yellow mosaic virus (CYMV), Cacao swollen shoot virus (CSSV), Sugarcane bacilliform virus (ScBV) and Taro bacilliform virus [5,6,7,8,9]. These have been used for a variety of purposes, including the investigation of infectivity and host range, symptom expression and virus localization in planta. Additional potential uses for infectious clones include their exploitation as virus-induced gene silencing (VIGS) vectors for plant functional genomics studies [10], as tools for foreign gene expression in plants and to study plant-virus interactions [11].

Cultivated bananas are hybrids between predominantly two wild *Musa* species, namely *Musa acuminata* (genome AA) and *M. balbisiana* (genome BB), which contribute the ‘A-genome’ and ‘B-genome’, respectively [12]. The genomes of hybrids can be diploid, triploid or tetraploid and comprised of A-only genomes (AA, AAA, AAAA) or can be A-B hybrids (AB, AAB, ABB) [13]. These variations in ploidy level, and the contributions of many *M. acuminata* subspecies, has resulted in hundreds of diverse banana cultivars being grown in subtropical and tropical regions of the world. These vary widely in their morphology as well as their use with, for example, distinct cultivars utilized as dessert, cooking and beer-making banana types [13].

A broad range of symptoms are reported from infections of bananas with BSVs. However, variations in the types of described symptoms, such as the shape and color of the leaf streaks, are often not categorically associated with a specified BSV species. The wide geographic occurrence of BSVs as well as the many different wild and cultivated banana subgroups, also complicates the association of specific symptoms with infection by distinct BSV species. Although BSV can be readily transmitted using mealybugs [2], the development of infectious clones of BSVs would simplify studies into their infectivity, host range and the association of specific symptoms with characterized BSV species/banana subgroups. In 2016, leaf tissue was collected from a dessert banana (*M. acuminata* cv. Dwarf Cavendish AAA) growing in a Brisbane suburban garden which was exhibiting chlorotic flecking symptoms typical of BSV infection. Here we report work to characterize an Australian isolate of Banana Streak CA Virus (BSCAV) isolated from the plant and demonstrate the infectivity of a cloned copy of the virus genome in a range of banana (*M. acuminata*) accessions. This is the first full-length BSCAV sequence from Australia, and the first report of an infectious clone of a BSV.

## 2. Materials and Methods 

### 2.1. Plant Material

The BSCAV isolate described here was collected from a Dwarf Cavendish banana plant growing in a suburban garden in Brisbane, Queensland, Australia, which was exhibiting typical symptoms of BSV infection. 

Tissue-cultured Williams (*Musa* AAA Cavendish subgroup) banana plants were provided by the Centre for Agriculture and the Bioeconomy, Queensland University of Technology, while tissue cultured plants of an additional eight banana genotypes were obtained from the Department of Agriculture and Fisheries, Queensland. These included four wild diploid AA accessions, namely *M. acuminata* subsp. *malaccensis* (Malaccensis), *M. acuminata* subsp. *siamea* (Khae Phrae), *M. acuminata* subsp. *truncata* (Truncata) and *M. acuminata* subsp. *zebrina* (Zebrina), as well as four cultivated banana accessions, namely Gros Michel (*Musa* AAA Gros Michel subgroup), Paka (*Musa* AA subgroup), Igisahira Gisanzwe (*Musa* AAA East African Highland Banana (EAHB) subgroup) and Pisang Madu (*Musa* AA subgroup). Williams plants were previously indexed for banana bunchy top virus (BBTV), while the other eight genotypes included were indexed for BBTV, cucumber mosaic virus, banana mild mosaic virus, banana streak virus and banana bract mosaic virus. Plants were multiplied in tissue culture and subsequently grown in rooting media as previously described [14]. 

### 2.2. Rolling Circle Amplification 

Total nucleic acid (TNA) was extracted from the leaves and subjected to RCA as described previously [15]. RCA-amplified DNA was independently digested using a range of restriction endonucleases (New England Biolabs, Australia) and analyzed by gel electrophoresis in 1% agarose gels in TBE buffer and stained with SYBR^TM^ Safe (ThermoFisher Scientific, Brisbane, Australia). 

### 2.3. PCR Detection of BSCAV

PCR for the detection of BSCAV was carried out using either primers Cav-F1/R1 [16] which amplify a 782 bp region of the genome including the 3′ end of ORF 3 and the 5′ 251 nt of the intergenic region, or primers Cav-1 (ATCCTTCTTTGGTTGACTCG) and Cav-12 (ATGAGTAATACGGTGACCAA) which amplify a 342 bp region of ORF 2. Briefly, 1 μL of TNA was mixed with 10 μL of 2x GoTaq Green Master Mix (Promega, Australia), and 5 pmol of each primer in a 20-μL reaction volume. PCR cycling conditions included an initial denaturation step at 94 °C for 3 min followed by 35 cycles of 94 °C for 30 s, 50/55 °C for 30 s for the Cav-F1/R1 or Cav-1/12 primers respectively, and 72 °C for 1 min and a final extension step at 72 °C for 5 min. Amplicons were separated by electrophoresis through 1.5% agarose gels in TBE and stained as above. As a positive control, TNA from a known BSCAV-infected banana sample (Ke171) [17] was included, while TNA from a healthy tissue cultured Cavendish banana plant was included as a negative control.

### 2.4. Cloning and Sanger Sequencing of the BSCAV-Brisbane Genomic Sequence

Partial and putative full-length genomic fragments resulting from *Hind*III or *Xba*I digestion, respectively, were ligated into appropriately digested, alkaline phosphatase-treated pUC19 and purified plasmid DNA was sequenced using an M13R primer, or in a primer-walking approach using sequence-specific primers. To confirm the putative single *Xba*I site in the full-length genome sequence, PCR was carried out on RCA product using sequence-specific primers and the amplicons cloned into pGEM^®^-T Easy (Promega, Alexandria, Australia) and sequenced using universal M13F/R primers. A list of the primer sequences used to generate the full-length genome of BSCAV-Brisbane is provided in Supplementary Table S1.

Sequencing was carried out using the Big Dye^®^ Terminator v3.1 Cycle Sequencing Kit (ThermoFisher Scientific, Australia) at the Molecular Genetics Research Facility, Queensland University of Technology, as per the manufacturer’s recommendations.

### 2.5. Construction of the Infectious Clone

**Figure 1:**
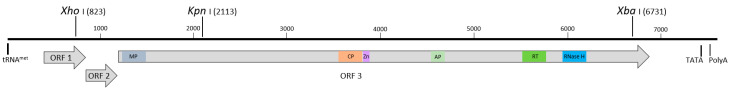
Linearized schematic representation of the genome organization of the BSCAV-Brisbane complete genome sequence. Block arrows represent the three open reading frames (ORFs) predicted from sequence analysis. Conserved protein motifs present in ORF 3 are indicated in color including the movement protein (MP, dark grey), coat protein (CP, orange), zinc finger domain (Zn, purple), aspartate proteinase (AP, light green), reverse transcriptase (RT, dark green) and ribonuclease H (blue). Additional genomic features highlighted include the conserved transfer RNA methionine (tRNA^met^) complementary sequence denoting the start of the viral genome, and the putative TATA and polyA sequences predicted from sequence analysis. The location of the three unique restriction sites *Xho*I, *Kpn*I and *Xba*I used for construction of the infectious clone is also shown.

To generate a greater-than-full-length cloned copy of the BSCAV genome, RCA products were subjected to two separate double-digestions. Firstly, *Xba*I and *Kpn*I were used to digest the genome within ORF 3 at the 3′ and 5′ ends, respectively, to generate two fragments of 4618 nt and 2819 nt. The ~3 kb fragment comprising the last 230 nt at the 3′ end of ORF 3, the complete IR, ORF 1, ORF 2, and the first 666 nt of ORF 3 (Figure 1) was excised from the gel following electrophoresis. Secondly, *Kpn*I and *Xho*I were used to generate respective fragments of 6147 nt and 1290, with the ~6 kb fragment comprising 4848 nt of ORF 3, together with the IR and the first 307 nt of ORF 1. This fragment (Figure 1) was also obtained by electrophoresis and gel-excision. The binary vector pOPT-EBX [18], which contains a multiple cloning site and an *nptII* plant selection cassette, was then double-digested using *Xba*I and *Xho*I and dephosphorylated. The two BSCAV genomic fragments were ligated into the linearized binary vector resulting in a terminally redundant molecule of 8966 nt (~1.2 × genome length). This vector, designated pOPT-EBX-BSCAV, was electroporated into *Agrobacterium tumefaciens* strain AGL1 and used for plant inoculation studies. An overview of the strategy used to prepare the infectious clone is provided in Supplementary Figure S1.

### 2.6. Plant Inoculation and Screening for Virus Infection

*A. tumefaciens* cultures were prepared as described previously [9]. To test the infectivity of pOPT-EBX-BSCAV, tissue-cultured banana plants, generated from virus-indexed mother plants, were acclimatized and planted into 10 cm pots in premium potting mix (Searles, Australia). Plants were grown in an enclosed plant house facility with a 12 h photoperiod at 25 °C. Plant inoculation was carried out by injecting the base of the pseudostem, approximately 2 cm above the soil level, with *Agrobacterium* containing the pOPT-EBX-BSCAV plasmid. To confirm the presence of BSCAV, TNA extracts were prepared as described above, or DNA extracts were prepared using the DNAzol^®^ reagent (ThermoFisher Scientific, Australia) as per the manufacturer’s instructions. Plants were tested at eight weeks post-inoculation using either RCA or PCR as described previously using 1−2.5 µL of TNA extract. For the detection of residual *Agrobacterium*, primers VCF/R, targeting the virC operon [19] were used, essentially as described above, but with an annealing temperature of 60 °C.

### 2.7. Transmission Electron Microscopy

Transmission electron microscopy (TEM) was carried out with partially purified virions prepared essentially as described by Lockhart [20]. Briefly, approx. 700 mg of leaf lamina/midrib tissue was taken from the youngest fully expanded symptomatic leaf of three Cavendish (Williams-AAA) bananas six weeks post-inoculation with pOPT-EBX-BSCAV. The pooled tissue was ground in 6 mL potassium phosphate buffer [0.2 M potassium phosphate pH 7, 15 mM EDTA, 2% PVP-4000, 2% PEG-8000, and 0.4% Na_2_SO_3_] together with acid-washed sand using a mortar and pestle. The homogenized samples were filtered through muslin into 10 mL ultracentrifuge tubes and centrifuged at 10,000 rpm for 15 min at 4 °C in a Beckman Optima L-90K Ultracentrifuge. Supernatants were decanted into fresh 10 mL tubes followed by the addition of 120 μL of 33% Triton X-100 and vigorous mixing for 1 min. Virions were pelleted by ultracentrifugation through a 30% sucrose cushion in 0.2 M potassium phosphate buffer pH 7.0, at 50,000 rpm for 30 min at 4 °C using a Beckman Optima L-90K Ultracentrifuge. Pellets were resuspended in 100 μL of 10 mM potassium phosphate buffer pH 7, transferred to 1.7 mL microcentrifuge tubes and organic extraction carried out by the addition of 30 μL of chloroform and vigorous mixing for 30 s. Extracts were then centrifuged for 5 min at 14,000 rpm in a benchtop microfuge and 75 μL of the supernatant transferred into new 1.7 mL microcentrifuge tubes. An aliquot (5 μL) of the partially purified virion preparation was then applied to electron-microscopy grids for 30 s at room temperature. Excess liquid was removed using filter paper and particles were subsequently stained using 2% ammonium molybdate pH 6.8 for 30 s at room temperature. Grids were examined using a JEOL-1400 120 kV transmission electron microscope at the Central Analytical Research Facility (CARF) Microscopy Laboratory, QUT.

## 3. Results and Discussion

### 3.1. Identification of BSCAV and Characterization of the Full-Length Sequence

TNA was extracted from the original banana leaf showing typical BSV symptoms and subjected to RCA. The RCA-amplified DNA was independently digested using *Bam*HI, *Eco*RI, *Hind*III, *Pst*I, *Xba*I and *Xma*I, with digestion using *Bam*HI or *Hind*III generating two fragments of ~7 and 1 kb, and ~5 and 3 kb, respectively, while digestion using *Eco*RI generated four to five distinct fragments of less than 2 kb. A single putative full-length fragment of ~8 kb was obtained following digestion with *Pst*I and *Xba*I, while digestion with *Xma*I did not produce any obvious digest fragments. Based on a comparison of these restriction profiles with published full-length BSV sequences in GenBank, the virus was tentatively identified as BSCAV. To confirm the presence of BSCAV, PCR was carried out with primers Cav-F1/R1, with the expected amplicon of 782 bp generated from both the positive control (Ke171) and Brisbane samples, but not from a healthy control TNA sample.

To further characterize the putative BSCAV isolate, the ~3 kb *Hind*III fragment obtained following digestion of the initial RCA reaction was cloned into pUC19 and five clones were sequenced using primer M13R. The five clones generated identical 940 nt sequences which showed 96.28% and 94.37% identity to Kenyan and French BSCAV isolates [17,21], respectively, and 84.03% identity to an unpublished isolate of ScBV from China (Accession no. KJ624754). To obtain the complete nucleotide sequence, the ~8 kb putative full-length badnavirus genome fragment generated following *Xba*I-digestion was cloned into pUC19 and three independent clones were sequenced in both directions. Sequences generated from the three independent clones were identical. The nucleotide sequence flanking the *XbaI* site was subsequently confirmed by PCR amplification with sequence-specific primers, followed by cloning and sequencing.

The complete genome comprised 7437 nt and contained three ORFs typical of badnaviruses and consistent with previously published BSCAV full-length sequences (Figure 1). ORFs 1, 2 and 3 comprised 531, 405 and 5514 nt, respectively, and encoded respective putative proteins of 21 kDa, 14.5 kDa and 212.5 kDa. Whereas the ORF 1/2 junction was represented by a 4 nt overlapping sequence (GGATGAGT), the ORF 2/3 junction overlapped by only a single nucleotide (TGATG). The intergenic region of 992 nt contained a typical 18 nt sequence, designated as the start of the genome, with 94.4% identity to plant tRNA^met^ as well as putative TATA box and polyA sequences consistent with previous reports [15,22]. BlastN analysis of the 579 nt RT/RNase H region delineated by the BadnaFP/RP primers [23] showed between 93.73% and 97.41% identity to BSCAV isolates with the highest identity to a Kenyan isolate (KF545100). Interestingly, a number of additional sequences with greater than 80% identity in the RT/RNase H region used for taxonomic purposes were identified, including between 93.32% and 97.17% identity to viruses belonging to ScBV ‘subgroup C’ [24], up to 82.19% for ScBV isolates related to Sugarcane bacilliform Guadeloupe A virus [24], as well as BSV isolates from Kenya (81.65%; KF545099) and Mexico (81.62%; KM259633). Comparison of the complete genome sequence showed 94.14 and 96.07% identity to the two published full-length BSCAV sequences in GenBank and approximately 80% nt identity with ScBGAV. This finding is consistent with previous work showing a close relationship between some sequences described from banana and sugarcane. Interestingly, with an increase in the number of published sequences now available in public databases, there are now some virus isolates with nucleotide sequence similarities in the RT/RNase H region which are higher than the current threshold of 80% set for distinct species in the genus *Badnavirus*. As such, a revision of the current threshold level is perhaps now warranted. 

### 3.2. Development of an Infectious Clone and Assessment of Infectivity in M. Acuminata Bananas

**Figure 2:**
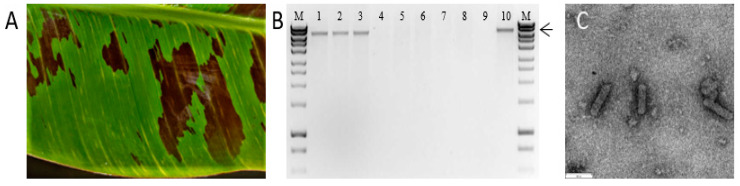
Inoculation of Cavendish (Williams-AAA) bananas with pOPT-EBX-BSCAV. (**A**) Typical chlorotic leaf streak symptoms of BSV infection at five weeks post-inoculation. (**B**) Rolling circle amplification analysis of TNA extracts prepared from inoculated and non-inoculated control plants at eight weeks post-inoculation. Lane M shows molecular weight (MW) marker Hyperladder 1 (Bioline, Australia), lanes 1–3 are extracts from pOPT-EBX-BSCAV inoculated plants, lanes 4–6 show plants inoculated with the binary vector pOPT-EBX only, lanes 7–8 are extracts from non-inoculated control plants, lane 9 is a no template control and lane 10 is the positive control. Arrow indicates the 8 kb MW marker fragment. The presence of a distinct ~8 kb fragment in plants inoculated with pOPT-EBX-BSCAV, but not in the control plants, is indicative of the detection of episomal viral DNA using RCA. (**C**) Transmission electron micrograph showing particles of BSCAV prepared from the leaves of banana plants six weeks post-inoculation with pOPT-EBX-BSCAV. Bar = 100 nm.

To test the infectivity of pOPT-EBX-BSCAV, three Cavendish (Williams-AAA) banana plants were initially agroinoculated using pOPT-EBX-BSCAV while, as controls, three plants were inoculated with an empty pOPT-EBX vector, and two plants were kept as non-inoculated controls. Plants were monitored for eight weeks post-inoculation. By five weeks post-inoculation, typical BSV symptoms (Figure 2A) developed on all three plants inoculated with pOPT-EBX-BSCAV, while the non-inoculated and empty vector control plants remained symptomless. At eight weeks post-inoculation leaf tissue was collected from all plants by sampling the youngest fully expanded leaf, TNA was extracted, and DNA amplified by RCA as described previously. RCA products were digested with *Xba*I and a single ~8 kb fragment was observed in digests from the three plants inoculated with pOPT-EBX-BSCAV, while no visible digest fragments were observed using TNA from the control plants (Figure 2B). To verify the RCA product from the banana plants inoculated with pOPT-EBX-BSCAV was not derived from residual *Agrobacterium*, TNA from each plant was tested for the presence of *Agrobacterium* by PCR and all samples tested negative (data not shown). Further, the ~8 kb *Xba*I digest fragment obtained from one banana plant inoculated with pOPT-EBX-BSCAV was cloned into pUC19 and subjected to Sanger sequencing. The nucleotide sequences obtained from the original source plant and the plant inoculated with pOPT-EBX-BSCAV were identical. Further, TEM of partially-purified virion preparations prepared from pooled samples from three plants inoculated with the BSCAV infectious clone revealed the presence of bacilliform-shaped virus particles of ~130 × 30 nm, typical of badnaviruses, (Figure 2C), while no virus-like particles were observed in extracts prepared from two non-inoculated control plants.

**Figure 3:**
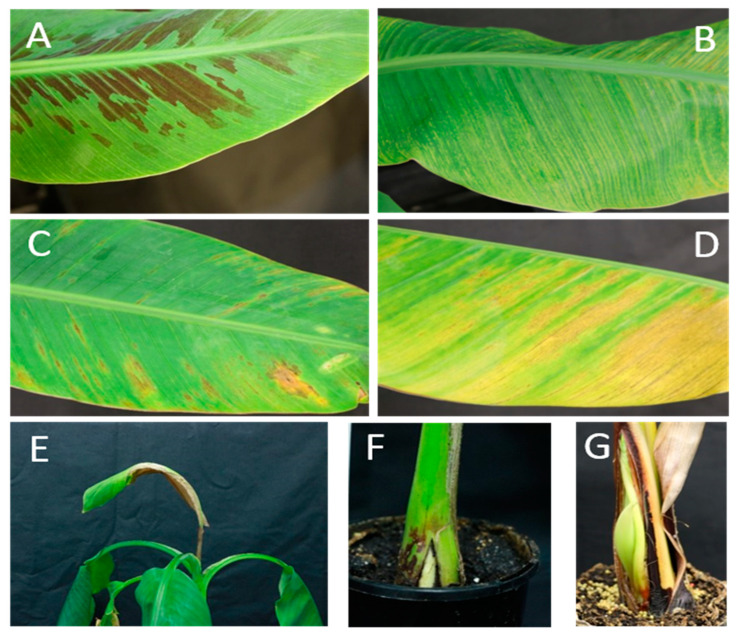
Inoculation of *Musa acuminata* bananas with pOPT-EBX-BSCAV. (**A**) Williams (Cavendish-AAA) leaf at 8 weeks showing typical mild flecking symptoms. (**B**) Pisang Madu (*Musa* AA subgroup) leaf at 8 weeks showing chlorotic streaking. (**C**) Truncata (*M. acuminata* subsp. *Truncata* (AA)) and (**D**) Igisahira Gisanzwe (*Musa* AAA-East African Highland Banana subgroup) leaves at 8 weeks showing chlorotic and necrotic areas. (**E**,**F**) Khae Phrae (*M. acuminata* AA subsp. *siamea*) plants at 16 weeks showing cigar leaf necrosis and pseudostem splitting. (**G**) Igisahira Gisanzwe plant at 28 weeks showing abnormal pseudostem growth/splitting.

The infectivity of pOPT-EBX-BSCAV in a range of banana genotypes was subsequently investigated using a diverse group of *M. acuminata* A-genome accessions (Table 1). Four-weeks old, acclimatized tissue-cultured banana plants were inoculated, plants were monitored for 28 weeks for typical symptom development, and all plants were tested for the presence of BSCAV by PCR at eight weeks post-inoculation. By four weeks post-inoculation, all five plants of Williams, Gros Michel, Paka, Pisang Madu and Malaccensis had developed virus-like symptoms, while four plants of Truncata and Khae Phrae and three plants of Igisahira Gisanzwe had developed symptoms. No obvious symptoms were observed on any Zebrina plants. Symptoms on most plants consisted of mild flecks typical of BSV (Figure 3A), however, the initial symptoms were noticeably stronger on Williams, Pisang Madu and Igisahira Gisanzwe. At eight weeks post-inoculation, all five Zebrina plants remained symptomless, while the three symptomatic Igisahira Gisanzwe plants had developed large, yellow-brown chlorotic areas as well as white, yellow and brown streaks (Figure 3D). In the four Khae Phrae plants with symptoms, yellow chlorotic flecks and streaks were very pronounced, while one plant also had necrosis of the cigar leaf (Figure 3E). All plants of the remaining six accessions showed symptoms by eight weeks post-inoculation (Table 1). Symptoms on Williams plants included mild yellow-green streaks, which were similar but milder in Gros Michel, Paka and Malaccensis plants and concentrated more towards the leaf margin and distal ends. Pisang Madu plants showed yellow chlorotic flecking symptoms which were more evenly distributed across the leaf lamina from the midrib to the margin (Figure 3B). Similarly, Truncata plants showed yellow and yellow-brown streaks extending from the midrib to the margin of the leaves (Figure 3C). In Paka, Pisang Madu and Gros Michel plants the symptoms were milder on the leaves which emerged after initial symptoms were seen around three to four weeks post-inoculation (Supplementary Figure S2).

To confirm the presence of BSCAV and the absence of *Agrobacterium*, leaf tissue was collected from all plants at eight weeks post-inoculation, DNA extracted, and PCR carried out using primers Cav-1/12. The expected size product of 342 bp was amplified from all the symptomatic plants confirming the presence of BSCAV (results not shown). Interestingly, all five symptomless Zebrina plants also tested positive for BSCAV, together with one additional plant of Igisahira Gisanzwe which was symptomless. Further, no products were generated using primers designed to amplify the virC gene indicating the absence of residual *Agrobacterium*.

All plants were monitored for an additional 20 weeks to observe changes in symptom development over time. The Zebrina plants remained asymptomatic throughout the experiment. In plants of Williams, Gros Michel, Malaccensis and Truncata, most of the newly emerging leaves were asymptomatic or had mild flecking symptoms, although in Gros Michel strong flecking/chlorosis symptoms could still be observed in one of the five plants at 28 weeks (Supplementary Figure S2). In Truncata, two plants showed clear chlorotic and/or necrotic flecking at 16 weeks, but the symptoms in all plants became mild yellow-brown flecks by 28 weeks (Supplementary Figure S2). New leaves in Pisang Madu and Paka plants typically also had milder symptoms than initially seen, however one plant of each genotype continued to show strong yellow flecking. By 28 weeks, leaves on plants from both genotypes again showed clear yellow flecking covering large areas of the leaf lamina (Supplementary Figure S2). 

In contrast, plants of both Khae Phrae and Igisahira Gisanzwe developed more severe symptoms over time. All four Khae Phrae plants which initially showed symptoms appeared to have stopped growing by 10 weeks post-inoculation, with two developing cigar leaf necrosis and an additional plant showing pseudostem splitting (Figure 3F). Eventually, all five plants developed symptoms, with 3/5 of the initial plants dying and suckers emerging which also developed severe symptoms. All five plants of Igisahira also eventually developed severe symptoms, including chlorosis and necrosis of the leaf lamina, as well as pseudostem splitting (Figure 3G).

In summary, the cloned copy of the BSCAV genome was shown to be highly infectious in all nine *M. acuminata* type bananas tested. By eight weeks post-inoculation, 43/45 plants tested positive using PCR, with all of these showing typical symptoms of BSV infection except those of Zebrina (*M. acuminata* AA subsp. *zebrina*) and one plant of Igisahira Gisanzwe which subsequently developed symptoms. Further, by the end of the experiment all plants from eight of the nine *Musa* accessions tested had developed symptoms of BSV infection. These results are consistent with previous reports describing the high infectivity of cloned badnavirus genomes, including CoYMV [5], CSSV [7], CYMV [8] and TaBV [9].

BSVs are known to cause a range of different symptoms in banana accessions. In this study, eight of the nine banana accessions tested developed typical BSV leaf streak symptoms, including yellow continuous or discontinuous flecking following inoculation with a BSCAV infectious clone. However, these symptoms were not always present on all infected plants throughout the experiment, with the flecking symptoms becoming milder in most accessions, and new leaves in some cases alternating between the presence or absence of symptoms. Interestingly, in Khae Phrae (*M. acuminata* AA subsp. *siamea*) and Igisahira Gisanzwe (*Musa* AAA-East African Highland Banana subgroup) plants, symptoms became more severe over time, resulting in necrosis of the leaves, stem splitting and even plant death in some cases. These results carried out under controlled growth conditions with BSCAV show that a range of distinct symptoms can be observed in response to infection with just one species of BSV. The observation that BSCAV could be detected in symptomless Zebrina plants shows that asymptomatic infections can also occur. Interestingly, infection of Zebrina with Banana streak MY virus was found to result in severe symptoms [14]. By preparing infectious clones of additional BSV species and inoculating these genotypes, the influence of *Musa* genotypes and virus species on symptom expression could be investigated further.

The results of this work show that the use of infectious clones of BSVs will facilitate studies investigating infectivity and symptom expression/variability in bananas. In addition, the synergistic effects of mixed infections, which have been observed under field conditions, could also possibly be investigated by co-inoculation of two or more infectious clones. Further work could also investigate the host range of both BSVs and ScBVs. Although ScBMOV has not been reported to naturally infect banana, agroinoculation of *M. acuminata* bananas with a cloned copy of the ScBV genome resulted in plants becoming infected [6]. In addition, rice, which is not a host plant of ScBV or BSV, also became infected when inoculated using the ScBV infectious clone, suggesting that further research on host range of badnaviruses will be possible with the availability of additional infectious clones.

## Figures and Tables

**Table 1:** Host range and symptoms of the BSCAV infectious clone in *Musa acuminata* bananas.

Accession Name (Taxonomic Subgroup) ^1^	No. of Plants Testing Positive by PCR at 8 Weeks Post-Inoculation	No. of Plants with Symptoms at 8 Weeks (Typical Symptoms)	No. of Plants with Symptoms at 28 Weeks (Typical Symptoms)
Williams (*Musa* AAA Cavendish subgroup)	5/5	5/5 (mild yellow-green streaks)	5/5 (mild yellow flecking or asymptomatic)
Gros Michel (*Musa* AAA Gros Michel subgroup)	5/5	5/5 (mild yellow-green streaks)	5/5 (mild yellow flecking or asymptomatic)
Malaccensis (*M. acuminata* AA subsp. *malaccensis*)	5/5	5/5 (mild yellow-green streaks)	5/5 (mild yellow flecking or asymptomatic)
Khae Phrae (*M. acuminata* AA subsp. *siamea*)	4/5	4/5 (yellow/chlorotic streaks; cigar leaf necrosis)	5/5 (cigar leaf necrosis; pseudostem splitting; plant death)
Truncata (*M. acuminata* AA subsp. *truncata*)	5/5	5/5 (continuous yellow and yellow-brown streaks)	5/5 (mild yellow-brown flecking or asymptomatic)
Zebrina (*M. acuminata* AA subsp. *zebrina*)	5/5	No symptoms	No symptoms
Paka (*Musa* AA subgroup)	5/5	5/5 (mild yellow-green streaks)	5/5 (yellow flecking and streaks)
Igisahira Gisanzwe (*Musa* AAA EAHB subgroup) ^2^	4/5	3/5 (yellow-brown chlorotic patches; white, yellow and brown streaks)	5/5 (chlorosis/necrosis of the leaf lamina; pseudostem splitting)
Pisang Madu (*Musa* AA subgroup)	5/5	5/5 (yellow/chlorotic flecking)	5/5 (yellow flecking and streaks)

^1^ See Musa Genome Information Service [25]. ^2^ EAHB—East African Highland Banana.

## Data Availability

The complete nucleotide sequence of the BSCAV isolate described in this study is available from GenBank with the accession number MW086553.

## Supplementary Materials

The following are available online at https://www.mdpi.com/article/10.3390/v13061071/s1, Figure S1: Overview of the preparation of an infectious clone of BSCAV, Figure S2: Symptoms of BSCAV-Brisbane infection in nine *Musa* genotypes under controlled conditions, Table S1: Sequence specific primers used to generate the full-length genome of the BSCAV isolate described in this study.
