# Supplementary material for: Infectivity of an Infectious Clone of Banana Streak CA Virus in A-Genome Bananas (Musa acuminata ssp.)
Source: Viruses. 2021 Jun 4;13(6):1071. doi: 10.3390/v13061071 (PMC8226583; doi:10.3390/v13061071)
Supplement: Supplementary file 1 [file viruses-13-01071-s001.zip › Supplementary Figure S1.pptx]

## Slide 1
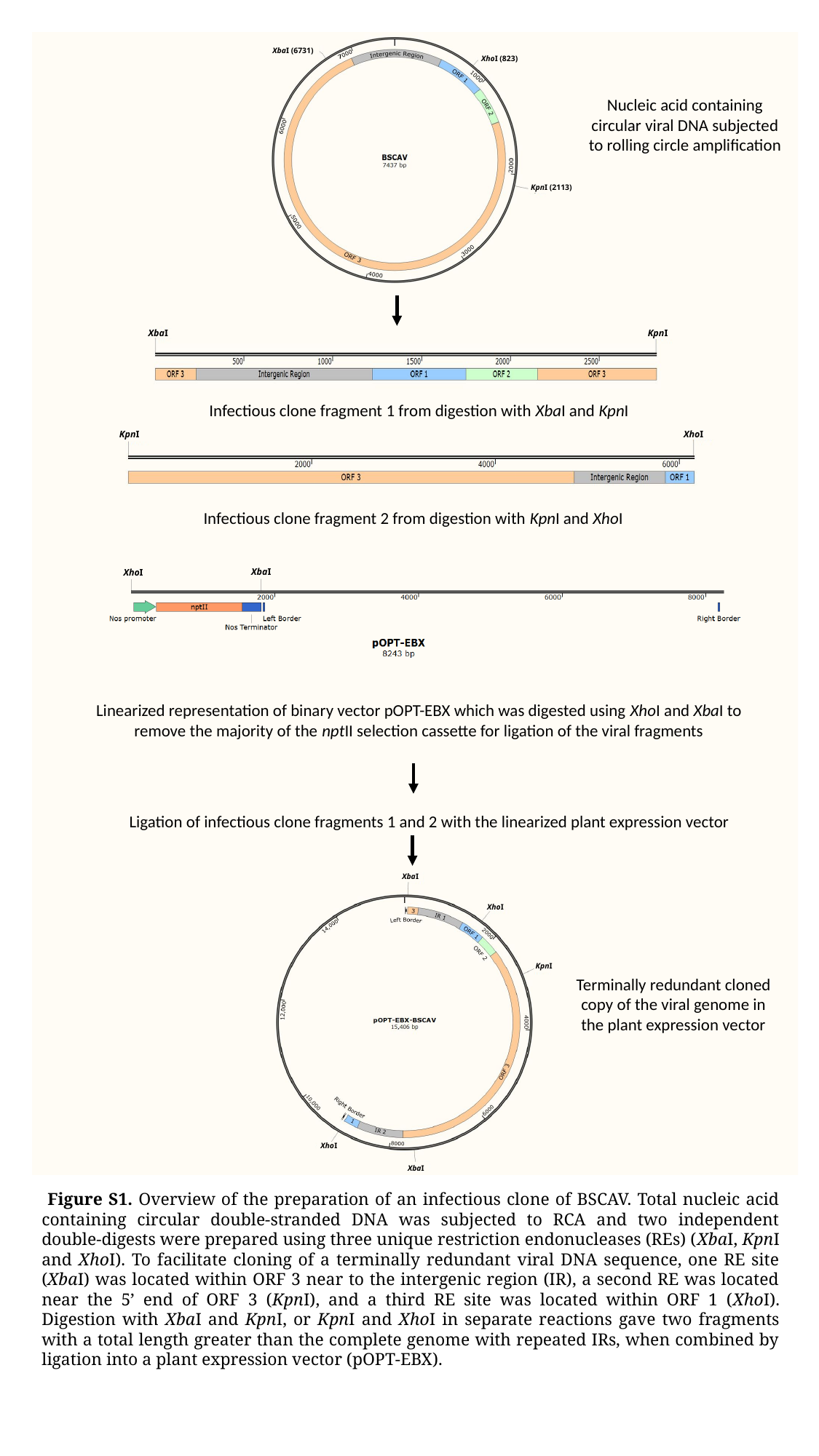

XbaI (6731)
XhoI (823)
Nucleic acid containing circular viral DNA subjected to rolling circle amplification
KpnI (2113)
XbaI
KpnI
Infectious clone fragment 1 from digestion with XbaI and KpnI
XhoI
KpnI
Infectious clone fragment 2 from digestion with KpnI and XhoI
XbaI
XhoI
Linearized representation of binary vector pOPT-EBX which was digested using XhoI and XbaI to remove the majority of the nptII selection cassette for ligation of the viral fragments
Ligation of infectious clone fragments 1 and 2 with the linearized plant expression vector
XbaI
XhoI
KpnI
XhoI
XbaI
Terminally redundant cloned copy of the viral genome in the plant expression vector
 Figure S1. Overview of the preparation of an infectious clone of BSCAV. Total nucleic acid containing circular double-stranded DNA was subjected to RCA and two independent double-digests were prepared using three unique restriction endonucleases (REs) (XbaI, KpnI and XhoI). To facilitate cloning of a terminally redundant viral DNA sequence, one RE site (XbaI) was located within ORF 3 near to the intergenic region (IR), a second RE was located near the 5’ end of ORF 3 (KpnI), and a third RE site was located within ORF 1 (XhoI). Digestion with XbaI and KpnI, or KpnI and XhoI in separate reactions gave two fragments with a total length greater than the complete genome with repeated IRs, when combined by ligation into a plant expression vector (pOPT-EBX).
