# Supplementary material for: Infectivity of an Infectious Clone of Banana Streak CA Virus in A-Genome Bananas (Musa acuminata ssp.)
Source: Viruses. 2021 Jun 4;13(6):1071. doi: 10.3390/v13061071 (PMC8226583; doi:10.3390/v13061071)
Supplement: Supplementary file 1 [file viruses-13-01071-s001.zip › Supplementary Figure S2.pdf]

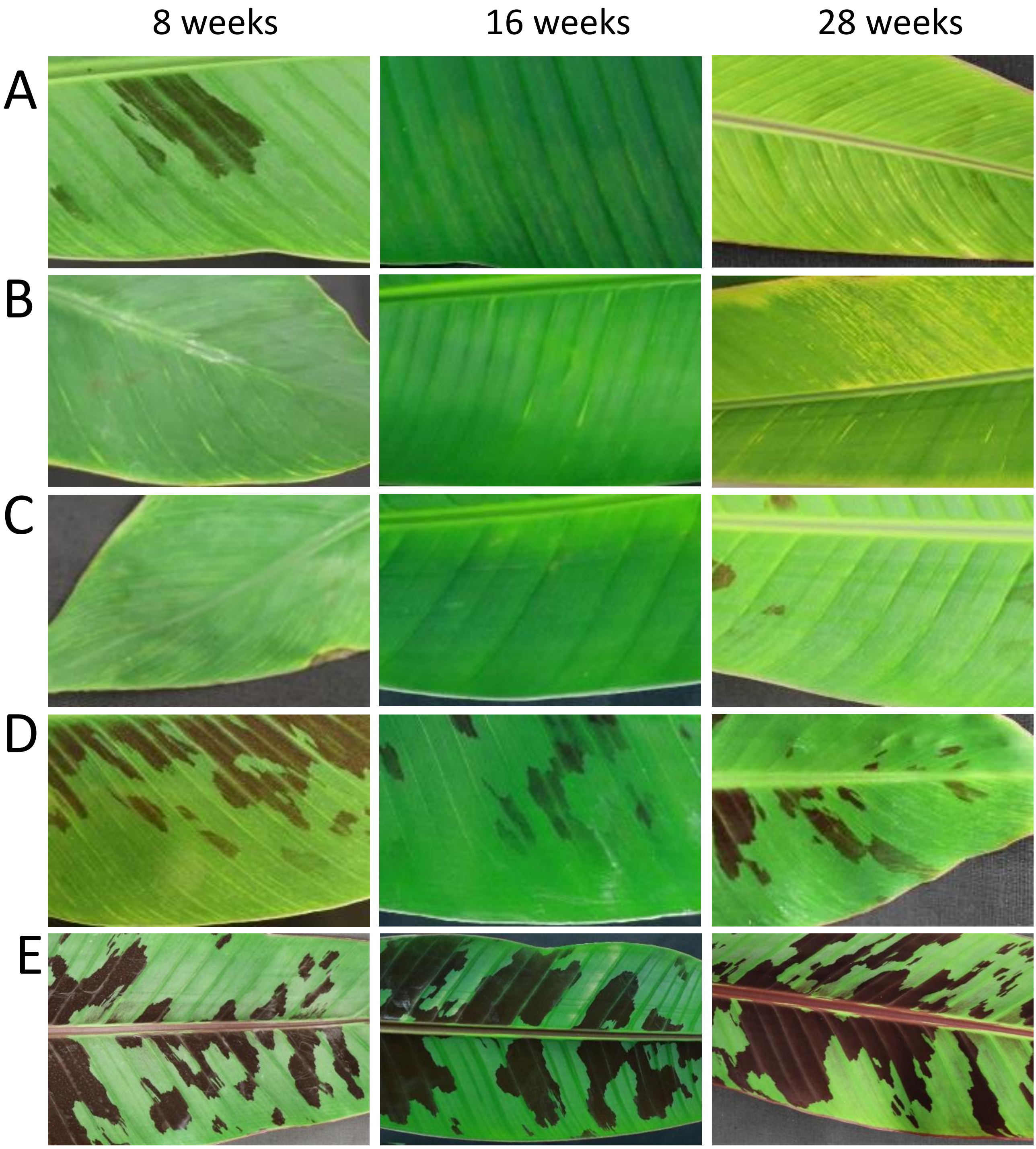
8 weeks
16 weeks
28 weeks
A
B
C
D
E

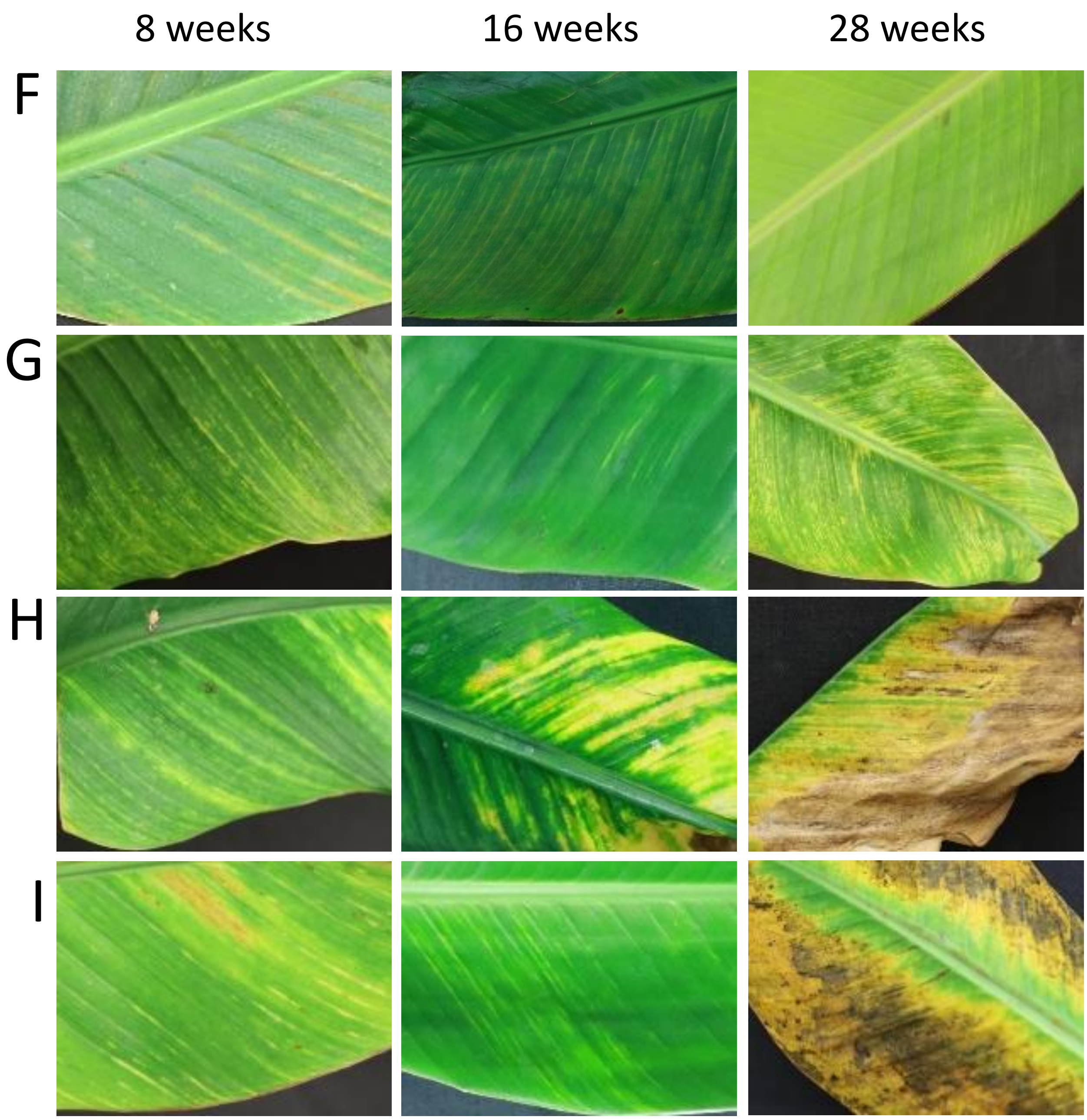

Figure S2. Symptoms of BSCAV-Brisbane infection in nine *Musa* genotypes under controlled conditions. Representative photos of symptoms at 8, 16 and 28 weeks post-inoculation with the BSCAV infectious clone. (A) Malaccensis (*M. acuminata* AA subsp. *malaccensis*) showing typical mild yellow-green streaks at 8 and 16 weeks, and yellow flecks which appeared by 28 weeks; (B) Paka (*Musa* AA subgroup) showing typical mild yellow-green streaks at 8 and 16 weeks, and the occurrence of yellow flecking/streaks at 28 weeks; (C) Gros Michel (*Musa* AAA Gros Michel subgroup) showing typical mild yellow-green streaks at 8 and 16 weeks; (D) Williams (*Musa* AAA Cavendish subgroup) showing typical yellow streaks at 8 weeks, which were milder at 16 and 28 weeks; (E) Zebrina (*M. acuminata* subsp. *zebrina*) leaves at 8, 16 and 28 weeks with no obvious symptoms of infection; (F) *M. acuminata* subsp. *truncata* showing continuous yellow-brown streaks at 8 weeks, continuous and discontinuous chlorotic streaks at 16 weeks and mild chlorotic streaks at 28 weeks; (G) Pisang Madu (*Musa* AA subgroup) showing yellow chlorotic flecking symptoms at 8 weeks, which were milder at 16 weeks and covered large areas of the leaf lamina at 28 weeks; (H) Khae Phrae (*M. acuminata* subsp. *siamea*) showing yellow/chlorotic streak symptoms on the leaves at 8 weeks, which coalesced into larger islands of chlorosis at 16 weeks, and subsequently resulted in severe chlorosis and necrosis by 28 weeks; and (I) Igisahira Gisanzwe (*Musa* AAA East African Highland Banana subgroup) showing yellow-brown chlorotic areas and streaks at 8 weeks, which in some cases

appeared milder and yellow-green at 16 weeks, but were more severe again by 28 weeks and resulted in chlorosis and necrosis of the leaf lamina.
