# Supplementary material for: Infectivity of an Infectious Clone of Banana Streak CA Virus in A-Genome Bananas (Musa acuminata ssp.)
Source: Viruses. 2021 Jun 4;13(6):1071. doi: 10.3390/v13061071 (PMC8226583; doi:10.3390/v13061071)
Supplement: Supplementary file 1 [file viruses-13-01071-s001.zip › Supplementary Table S1.pdf]

**Table S1:** Sequence specific primers used to generate the full-length genome of the BSCAV isolate described in this study

| Primer Name | Sequence 5′ – 3′ |
|---|---|
| BSV-Cav1 | ATCCTTCTTTGGTTGACTCG |
| BSV-Cav2 | AGCATTCACTTTCTCTTCCC |
| BSV-Cav3 | AACCAAATGGCATAACAAGC |
| BSV-Cav4 | GAATGAATAGGCAAGATTGG |
| BSV-Cav5 | ACTATAGTGGAGTTGGTACG |
| BSV-Cav6 | GATATTACGTCTTACTGGAG |
| BSV-Cav7 | CGCAGCTGAAGAAGAACAAG |
| BSV-Cav8 | AGAGCTAAAGGGAAAAGAGC |
| BSV-Cav9 | CAGTCCAGCAGCATAAAGAG |
| BSV-Cav10 | GGAAGGAGATAGCACTAAGG |
| BSV-Cav11 | ATAGAAAATAGTATCCCGGA |
| BSV-Cav12 | ATGAGTAATACGGTGACCAA |
| BSV-Cav13 | CTAGAACATCAAGAAATCCA |
| BSV-Cav14 | GAGAATGAGCTACTTAATGCC |
| BSV-Cav15 | GTTTTCTGATTCTCTCTTCTAGC |
| BSV-Cav16 | GCAGAAGAATTCATCGAGCC |
| BSV-Cav17 | CTAAGTCCAGTGAATCCAGG |
| BSV-Cav18 | AAGACAGGAAGAGCATGGGC |
| BSV-Cav19 | GAACTGGTTATTTCCAGCCC |
| BSV-Cav20 | CGCAAAGCGGTAGGTCCAGAGG |
| BSV-Cav21 | GTCATCATAATATCTGGGATG |
| BSV-Cav22 | CAACTGCCTTGTGATCTGGG |
| BSV-Cav23 | CGTACCTGGTCAGTCACAGAGG |
| BSV-Cav24 | ATCGGGGACTACCTCATCTGGC |
